# Health-related quality of life in children under treatment for overweight, obesity or severe obesity: a cross-sectional study in the Netherlands

**DOI:** 10.1186/s12887-023-03973-8

**Published:** 2023-04-11

**Authors:** Bibian van der Voorn, R. Camfferman, J. C. Seidell, J. Halberstadt

**Affiliations:** Faculty of Science, Department of Health Sciences, Section Youth and Lifestyle, Vrije Universiteit Amsterdam, De Boelelaan 1085, Amsterdam, 1081 HV The Netherlands

**Keywords:** Pediatric obesity, Overweight, Quality of life, Patient-reported outcome measures, Europe

## Abstract

**Background:**

It is unknown whether weight class is associated with impairment of health-related quality of life (HRQOL) for children in the Netherlands. The aim of this study was to explore generic and weight-specific HRQOL in a clinical cohort of children with overweight, obesity or severe obesity aged 5–19 years in the Netherlands.

**Methods:**

803 children from three clinical cohorts participated: mean age 11.5 (SD 2.9) years, 61.1% girls. The influence of weight class was explored in a subgroup of 425 children (25.2% with overweight, 32.5% obesity and 42.3% severe obesity), of whom the exact International Obesity Task Force (IOTF) BMI class was known. Generic HRQOL was measured by the PedsQL child report. Weight-specific HRQOL was measured by the IWQOL-Kids child or parent report. Average total, subscale and item scores were reported and the influence of the IOTF BMI class analyzed by multiple linear regression, corrected for age and sex.

**Results:**

Children with severe obesity had lower generic and weight-specific HRQOL scores than those with obesity or overweight. IOTF BMI class was negatively associated with item scores from all subscales, especially physical, social and emotional functioning. Children with overweight reported similar HRQOL total, subscale and item scores to children with obesity.

**Conclusions:**

In the Netherlands, children treated for overweight, obesity or severe obesity experience problems on the majority of items within all subscales of generic and weight-specific HRQOL. Children with severe obesity especially report significantly more challenges due to their weight than children with obesity or overweight.

**Supplementary Information:**

The online version contains supplementary material available at 10.1186/s12887-023-03973-8.

## Introduction

The prevalence of childhood overweight and obesity has reached epidemic proportions in many parts of the world [1]. Overweight and, even more, obesity and severe obesity, are associated with physical and psychological comorbidities, including impaired health-related quality of life (HRQOL) [2–4].

HRQOL measures can be used as patient-reported outcome measure (PROM) and have added value when screening for factors that hinder a healthy lifestyle, when designing a tailored intervention strategy, and when evaluating effectiveness of treatment [5, 6]. There are some inconsistencies in the definition of HRQOL that is used in literature [7, 8]. In this study, HRQOL reflects “those aspects of self-perceived well-being that are related to or affected by the presence of disease or treatment” [9]. HRQOL of children with overweight or obesity can be measured by at least ten validated questionnaires, all with their own strengths and limitations [2, 10]. Commonly used generic HRQOL measures for children with obesity are: KIDSCREEN, KINDL, and the Pediatric Quality of Life Inventory (PedsQL). In addition, weight-specific HRQOL measures available for children with overweight or obesity are: Sizing Me Up, Sizing Them Up, Health-related Quality of Life, Youth Quality of Life Instrument-Weight Module and the Impact of Weight on Quality of Life-Kids (IWQOL-Kids) [2, 10]. In the Netherlands, the PedsQL and the IWQOL-Kids are often used, due to its utility, feasibility and usability as HRQOL measure in children with overweight or obesity, living in the Netherlands [8]. Moreover, these questionnaires are often used in international literature, enabling comparison with other cohorts. The PedsQL is a validated measure, often used to compare children with overweight/obesity to children without overweight [11–13] or to children with other chronic diseases. The IWQOL-Kids, a validated measure, yields more information about weight-specific impairments and has a greater sensitivity in detecting changes in HRQOL as a treatment effect in children with overweight or obesity compared to questionnaires that assess only generic HRQOL [8, 9, 14–16].

Studies indicate that more severe levels of overweight/obesity are associated with lower levels of HRQOL. An association that seems stronger with increasing age (4–18 years) [17]. Children from clinical cohorts report lower HRQOL scores than children with overweight or obesity in population cohorts [2, 18]. The few studies that include children with severe obesity (BMI > 99th percentile) do confirm the negative association between HRQOL and weight class [2, 18–22]. Still, reported associations between HRQOL and BMI vary per cohort and study design, and are lacking in approximately 10% (3 out of 34) of publications [2].

Although the association between HRQOL and weight class has not yet been properly studied in a large clinical sample of children in the Netherlands, research indicates that this association is likely to be similar to that in other countries [23–29]. However, most of these studies investigated generic HRQOL and not weight-specific HRQOL, and reported only summary measures, i.e. total and subscale scores, instead of analyzing a combination of total, subscale and item scores. To adequately understand specific problems experienced by children and the development of tailored interventions to improve quality of life, insights at the item level may be of importance [8].

Overall, both generic and weight-specific HRQOL measures can be of great value when used as PROM, independently or synergistically. Therefore, in the current study the HRQOL will be explored within a relatively large clinical cohort of children with overweight, obesity or severe obesity between ages 5 and 19 in the Netherlands. Influence of severity of the obesity will be examined on the total, subscale and item scores of generic HRQOL and weight-specific HRQOL.

## Method

### Participants and procedure

A total of 803 Dutch children under treatment for overweight, obesity or severe obesity, aged between 5 and 19 years, participated in this study. Data collection was part of multiple studies: (1) baseline data of a large cohort study [25] (HELIOS, *N* = 120), in which children with severe obesity (ages 8–19) participated in an intensive inpatient treatment; (2) baseline data of an outpatient combined lifestyle intervention for children with all grades of overweight and obesity (ages 7–13) [30] (LEFF, *N* = 358); and (3) additional data collection via a newly developed webtool (*N* = 325) in the regular youth healthcare setting for children with overweight, obesity and severe obesity (ages 5–18). Altoghether, baseline data were collected from 27 healthcare settings throughout the Netherlands before starting treatment, between 2016 and July 2018.

All generic and weight-specific HRQOL measures were prospectively collected and used for clinical as well as research purposes. We tested the understanding of language and adequacy of the response for all children in cohort 1 and a representative subsample of cohort 3. In addition, a conversation after completion of the questionnaire was held with all children and parents from cohort 3. This conversation was used to both check the answers as well as interpret the information and subsequent include it in the clinical evaluation. This conversation took place independent of the child’s age, since all data for this cohort were collected for clinical care purposes. Ethical approval was given by the medical ethics committee of VU University Medical Center Amsterdam for the first two studies, HELIOS and LEFF. For the additional data collection full ethical approval was waived by the same medical ethics committee (VUMC Amsterdam), as data were collected for clinical care purposes. In all cases, data was only included when participants’ parents gave informed consent to use their scores for scientific purposes. Informed consent from children over age 12 themselves was also required, in addition to the consent of their parents.

### Measures

***Generic health-related quality of life.*** The Pediatric Quality of Life Inventory (PedsQL) 4.0 [31] is a validated measure to assess generic HRQOL. In this study the Dutch translation of the child self-report version for ages 8–12 and the teenager self-report version for ages 13–18 was used. The questionnaire consists of 23 items that can be divided into four scales: physical functioning, emotional functioning, social functioning and school functioning. The items were scored on a 5-point scale: 0 = never a problem, 1 = almost never a problem, 2 = sometimes a problem, 3 = often a problem, 4 = almost always a problem. Similarly to the original study [31], items were reverse-scored and linearly transformed to a 0–100 scale (0 = 100, 1 = 75, 2 = 50, 3 = 25, 4 = 0). A higher score represents a better generic HRQOL. The original PedsQL has a high reliability (Cronbach α on total score = 0.88, Cronbach α on subscales range between 0.68–0.90) and validity [32]. The reliability in our total sample for the PedsQL child report was good for the total scores (α = 0.84) and fairly good for the subscales (range α: 0.71–0.80).

***Weight-specific Quality Of Life.*** The Impact of Weight on Quality of Life for Kids (IWQOL-Kids) [14] is a validated measure to assess weight-specific HRQOL. Two versions were used: the child self-report and the parent proxy-report (Dutch translation [8, 27]). The IWQOL-Kids was developed and validated for the 11–19 age group[14]. Since reliability and validity is not known for younger children, we checked in practice the appropriateness of use for this group. Subsequently we choose to use the IWQOL-Kids also for children aged 5–11 in our observational study (parent proxy-report under age 7, and child- or parent proxy-report for ages 7–11), and data of this younger age group was included in the total analysis.

The questionnaire consists of 27 items, which can be divided into four subscales: physical comfort, body esteem, social life and family relations. The items were scored on a 5-point scale: 1 = always, 2 = mostly, 3 = sometimes, 4 = rarely, 5 = never. Similarly to the original study [14], the total and subscale scores were calculated as an unweighted sum of scores on the items, followed by transforming these scores on a 0–100 scale. A higher score represents better weight-specific HRQOL.

Due to differences in clinical protocols, the questionnaire used to assess weight-specific HRQOL differed: HELIOS used the IWQOL-Kids child-report for all participants [25], LEFF used the IWQOL-Kids parent proxy-report for all participants [30], and the protocol for the additional data collection via the webtool varied per location and therapist. See Table 1 for an overview of the sample sizes per cohort and questionnaire. As a result, only five paired child- and parent proxy-report scores were eligible for the IWQOL-Kids. Of these participants, we only included the child report.

**Table 1:** Collected data per cohort and questionnaire

	PedsQL child-report	IWQOL-Kids child-report	IWQOL-Kids parent proxy-report
**Cohort 1 (HELIOS)** N = 120	N = 119	N = 120	N/A
**Cohort 2 (LEFF)** N = 358	N = 345	N/A	N = 317
**Cohort 3 (Additional)** N = 325	N = 97	N = 198	N = 69*

* Different individuals than those that completed the IWQOL-Kids child-reports

The original IWQOL-Kids has a high reliability (Cronbach α on total score = 0.96, Cronbach α on subscales range between 0.88–0.95) and validity [14]. The reliability in our total sample (ages 5–19) was adequate for the total scores (child: α = 0.78, parent proxy α = 0.78) and varied for the subscale scores (child α range: 0.78–0.92, parent proxy α range: 0.67–0.89), with the subscale score for family relations having the lowest reliability (child α: 0.78, parent proxy α: 0.67).

***Weight class.*** Weight class was measured in 0.1 kg increments and height in 0.1 cm increments by professionals as part of routine care. Body mass index scores (BMI, kg/m^2^) were calculated, as were standard deviation scores-BMI (SDS-BMI) scores (BMI corrected for age and sex), according to the Dutch national growth references [33]. Overweight, obesity and severe obesity were based on the international (International Obesity Task Force, IOTF) BMI cut-off points of Cole and Lobstein TJ Cole and T Lobstein [34].

### Statistical analyses

Analyses were performed using SPSS version 27. First, the distribution of all continuous baseline and dependent variables was tested. Second, average total, subscale and item scores were calculated for the total group on the PedsQL child report and the IWQOL-Kids child- and parent proxy-report. While all dependent variables were parametric, mean (SD) scores were reported. Third, for children whose exact weight, height, age and sex were known (N = 425), linear regression was used to analyze the association with IOTF BMI class, corrected for age and sex. Fourth, multiple regression analyses with dummy coding of IOTF BMI classes were used to test weight class differences for the total and subscale scores. Fifth, a sensitivity analysis was done to test the robustness of our data: PedsQL scores for the age group ≥ 8 years were analyzed as well as IWQOL-Kids scores for the age group ≥ 11 years.

## Results

Because of the observational character of this study, baseline characteristics such as weight class, age and sex were not known for all participants. Table 2 summarizes the available data.

**Table 2:** Baseline characteristics of the total sample and per cohort

		All together N = 803	Cohort 1 (HELIOS) N = 120	Cohort 2 (LEFF) N = 358	Cohort 3 (Additional) N = 325
IOTF BMI class *	**Overweight (%)**	107 (25.2%)	N/A	99 (37.8%)	8 (17.4%)
	**Obesity (%)**	138 (32.5%)	7 (6%)	115 (43.9%)	16 (34.8%)
	**Severe Obesity (%)**	180 (42.3%)	110 (94%)	48 (18.3%)	22 (47.8%)
Age *	**Mean (SD) in years**	11.5 (2.9)	14.8 (2.4)	10.1 (1.5)	11.5 (3.3)
Sex*	**N (%) girls**	284 (61.1%)	81 (67.5%)	169 (62.6%)	34 (45.3%)

* IOTF BMI class was known for 425 children, age for 503 children and sex for 465 children. Percentage given reflects the valid percentage for the column per characteristic (within-cohort). Number of missing characteristics per cohort: HELIOS 3 out of 120; LEFF 96 out of 358; Additional 279 out of 325

### Generic HRQOL

***Child report.*** For each item, large groups of children scored ≥ 1, i.e. experiencing it to be a problem to some degree (range per item: 31.7–72%), with a substantial portion scoring ≥ 3, i.e. experiencing it to be a problem often or almost always (range per item 4.5–19.4%). Few children scored 0 on all items, i.e. reported not experiencing any items related to generic HRQOL to be a problem (*N* = 5, 1%).

Table 3 shows average scores on generic HRQOL items and whether a significant association with IOTF BMI class was present; all total scores and subscale scores (p < 0.05), as well as some of the generic HRQOL item scores within the physical and social functioning domains were negatively associated with IOTF BMI class (Bonferroni adjusted p < 0.0022).

**Fig. 1:**
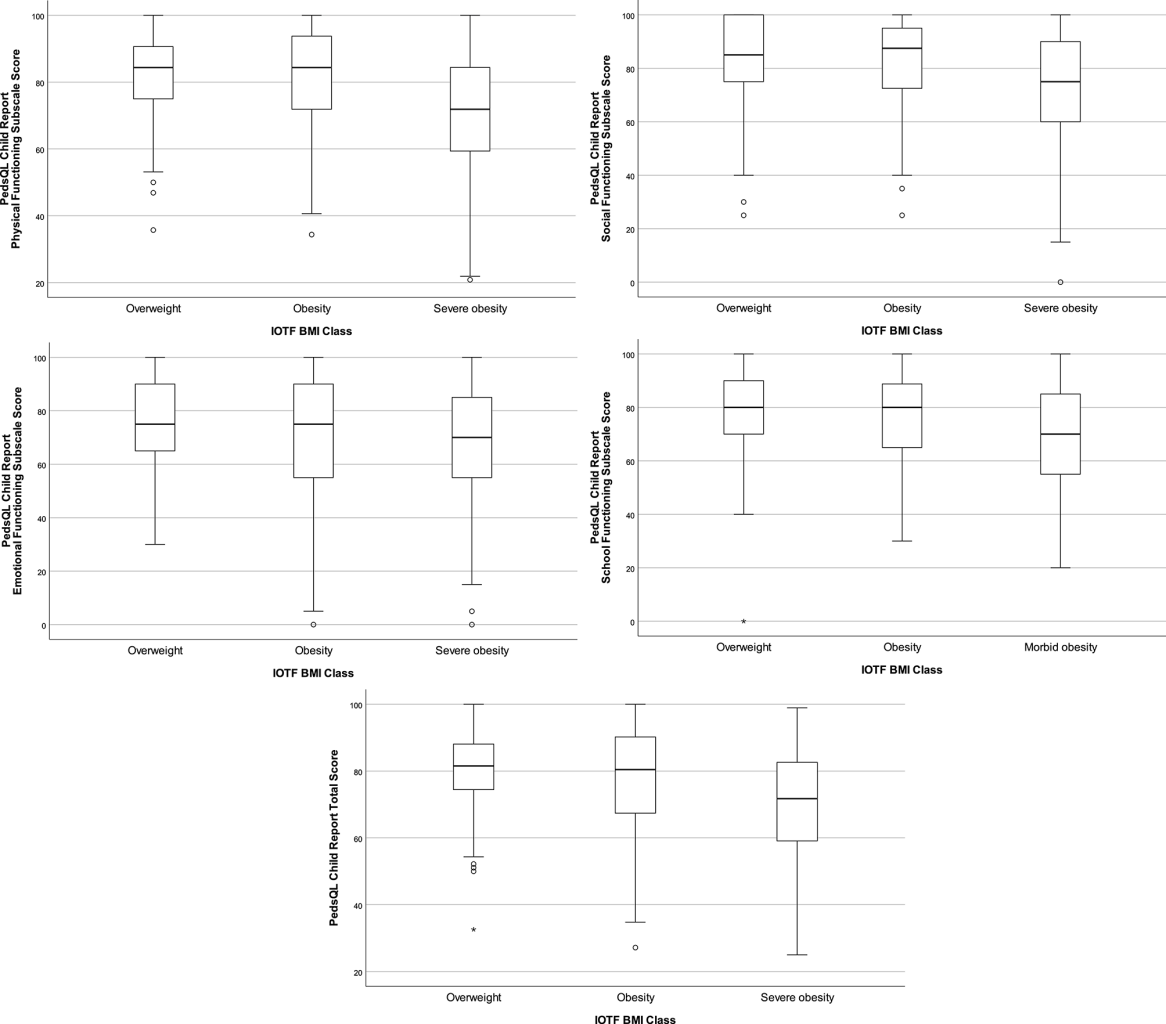
Boxplots of PedsQL child-report total and subscale scores per IOTF BMI class

Figure 1 and Supplementary Table 1 show the differences on total and subscale PedsQL child-report scores between IOTF BMI classes. All total and subscale scores were significantly lower for the group with severe obesity than for the group with overweight, adjusted for age and sex (p < 0.05). The total and subscale scores on physical and social functioning were also significantly lower for the group with severe obesity than for the group with obesity, adjusted for age and sex (p < 0.05). A trend (p < 0.10) toward a significantly lower score for school functioning was found for the group with severe obesity than for the group with obesity, adjusted for age and sex.

**Table 3:** Average total, subscale and item scores for the PedsQL child report (n = 561)

	Mean	SD	P-Value
**Physical functioning**	78.7*	**16.0**	**< 0.001**
1. Hard to walk more than one block	79.1*	28.8	< 0.001
2. Hard to run	72.2*	31.2	< 0.001
3. Hard to do sports or exercises	76.4*	27.1	< 0.001
4. Hard to lift something heavy	77.7	25.9	0.656
5. Hard to take bath or shower	93.5	17.5	0.469
6. Hard to do chores around the house	84.1	25.0	0.379
7. Hurth or aches	73.1	26.6	0.275
8. Low energy	73.5*	26.9	< 0.001
**Emotional functioning**	73.1*	**21.0**	**0.009**
9. Feel afraid or scared	79.8	23.8	0.152
10. Feel sad or blue	73.1	27.2	0.050
11. Feel angry	68.0	26.7	0.091
12. Trouble sleeping	70.6	33.6	0.028
13. Worry about what will happen	74.2	30.2	0.015
**Social functioning**	79.2*	**20.0**	**< 0.001**
14. Trouble getting along with peers	83.6	23.7	0.445
15. Other kids not wanting to be friend	82.5	25.5	0.036
16. Teased	78.0*	28.8	< 0.001
17. Doing things other peers do	69.8	28.3	0.004
18. Hard to keep up when play with others	81.8*	25.9	< 0.001
**School functioning**	74.4*	**18.7**	**0.005**
19. Hard to concentrate	77.7	27.2	0.357
20. Forget things	67.4	27.1	0.269
21. Trouble keeping up with schoolwork	75.2	29.3	0.011
22. Miss school – not well	78.6	25.1	0.032
23. Miss school – doctor appointment	73.1	27.2	0.010
**Total score**	76.7*	**15.3**	**< 0.001**

* Significant association (p < 0.05) with IOTF BMI class, adjusted for age and sex. For item scores a Bonferroni adjusted p- value 0.05/23 = 0.0022 was considered statistically significant

### Sensitivity analysis for the PedsQL

The reliability in the subset of our sample aged < 8 years (n = 23) was lower than that of the total sample (n = 561), except for the domain social functioning. The age was unknown of 174 children that completed the PedsQL, so these could not be included in this comparison. Differences in child-report for age < 8 vs. ≥ 8 are α: total score 0.78 vs. 0.85; physical functioning 0.66 vs. 0.77; emotional functioning 0.73 vs. 0.80; social functioning 0.89 vs. 0.80; school functioning: 0.61 vs. 0.73.

Supplementary Table 3 displays the absolute scores of the age group for which the PedsQL child-report was validated, i.e. ≥8 years. In this smaller sample (N = 363), scores are slightly lower. Statistical significance did not change.

### Weight-specific HRQOL

***Child-report.*** For each item in the weight-specific HRQOL questionnaire, large groups of children scored ≤ 4, i.e. reported that their weight impacted the item to some degree (range per item: 26–86%), except on the family scale items (9–28%). For each item, a substantial portion of the children scored ≤ 1, i.e. reported that their weight had always or most times impact on that specific item (range 2–49%). Few children scored 5 on all items, i.e. reported not experiencing any impact on weight-specific HRQOL (*N* = 5, 1.6%).

Table 4 shows average scores on weight-specific HRQOL items. Because data on the IWQOL-Kids child-report together with IOTF BMI class, age and sex were only known for 113 children with severe obesity, seven children with obesity and one child with overweight, we could not analyze the association between weight-specific HRQOL scores and IOTF BMI class.

30% or more of children with severe obesity reported that they are always or often ashamed of their body, lack self-confidence, have difficulty buying clothing, and don’t like undressing in front of others.

**Table 4:** Average total, subscale and item scores for the IWQOL-KIDS child-report (n = 318)

IWQOL-KIDS child-report	Mean	SD
**Physical comfort**	74.1	20.4
1. Avoid stairs	77.3	27.4
2. Hard to bend over	73.3	29.4
3. Hard to move around	72.7	28.0
4. Hard to fit into seats in public places	87.7	22.2
5. Knees or ankles hurt	71.5	30.0
6. Hard to cross legs	62.2	36.8
**Body esteem**	**60.8**	**26.98**
7. Ashamed of body	45.3	32.9
8. Don’t like myself	72.5	32.2
9. Try not to look at myself in mirrors or photographs	77.4	30.0
10. Hard time believing compliments	64.8	34.5
11. Lack in self-confidence	60.3	36.7
12. Avoid activities that involve wearing shorts/bathing suits	64.3	35.9
13. Difficult to buy clothing	42.3	34.9
14. Don’t like to undress in front of others	52.9	40.0
15. Embarrassed to try out for activities at school	67.6	35.1
**Social life**	**79.1**	**20.4**
16. Teased or made fun of	69.0	28.9
17. People talk about me behind my back	70.5	28.7
18. People avoid spending time with me	87.6	21.6
19. People stare at me	71.7	29.7
20. Trouble making/keeping friends	87.3	23.4
21. People think that I am not smart	88.7	22.4
**Family relations**	**92.9**	**12.3**
22. Family members treat me differently	88.7	21.8
23. Family member talk behind my back	92.4	17.9
24. Family members reject me	96.2	14.0
25. My parents are not proud of me	93.5	18.6
26. Family members make fun of me	90.6	18.8
27. Family members don’t want to be seen with me	96.1	14.8
**Total score**	**75.0**	**17.0**

**Table 5:** Average total, subscale and item scores for the IWQOL-KIDS parent proxy-report (n = 385)

IWQOL-KIDS parent proxy-report	Mean	SD	P-Value
**Physical comfort**	85.2*	16.5	< 0.001
1. Avoid stairs	82.1	27.1	0.052
2. Hard to bend over	80.4	27.3	0.014
3. Hard to move around	83.1**†**	45.9	0.003
4. Hard to fit into seats in public places	94.9*	16.0	< 0.001
5. Knees or ankles hurt	87.4*	21.5	< 0.001
6. Hard to cross legs	84.9*	25.1	< 0.001
**Body esteem**	79.3*	**20.2**	0.018
7. Ashamed of body	69.6	29.8	0.354
8. Don’t like myself	84.9	23.5	0.284
9. Try not to look at myself in mirrors or photographs	89.4	21.6	0.021
10. Hard time believing compliments	84.5	26.2	0.060
11. Lack in self-confidence	78.1	28.6	0.269
12. Avoid activities that involve wearing shorts/bathing suits	84.7	26.4	0.026
13. Difficult to buy clothing	65.3	33.2	0.020
14. Don’t like to undress in front of others	72.7	33.5	0.534
15. Embarrassed to try out for activities at school	84.2	24.9	0.005
**Social life**	86.4*	**15.9**	< 0.001
16. Teased or made fun of	76.7	25.8	0.009
17. People talk about me behind my back	79.3*	25.3	0.001
18. People avoid spending time with me	90.7**†**	18.6	0.003
19. People stare at me	87.1*	20.9	< 0.001
20. Trouble making/keeping friends	90.6*	18.9	< 0.001
21. People think that I am not smart	93.8*	14.6	0.001
**Family relations**	94.5*	**9.0**	**0.008**
22. Family members treat me differently	92.4	17.6	0.135
23. Family member talk behind my back	92.7	17.1	0.128
24. Family members reject me	97.8	9.1	0.019
25. My parents are not proud of me	96.7	13.4	0.130
26. Family members make fun of me	88.7	19.9	0.081
27. Family members don’t want to be seen with me	98.8	8.3	0.017
**Total score**	85.5*	**12.9**	< 0.001

* Significant association with IOTF BMI class, adjusted for age and sex (p ≤ 0.05). For item scores a Bonferroni adjusted p- value 0.05/27 = 0.0019 was considered statistically significant

**†** Trend toward a significant association with IOTF BMI class, adjusted for age and sex (Bonferroni adjusted p ≤ 0.10/27 = 0.0037)

***Parent proxy-report***. Scores on the weight-specific HRQOL (N = 390) reported by parents of children with overweight/obesity/severe obesity were comparable with child-report scores (see Table 5). On all items, parents scored ≤ 4, i.e. reported that their child with overweight/obesity/severe obesity experienced that their weight has impact on the item to some degree (range 3–63%). For each item, a substantial portion of the parents scored ≤ 1, i.e. reported that their child’s weight has impact on the item often or almost always (range 1–23%).

**Fig. 2:**
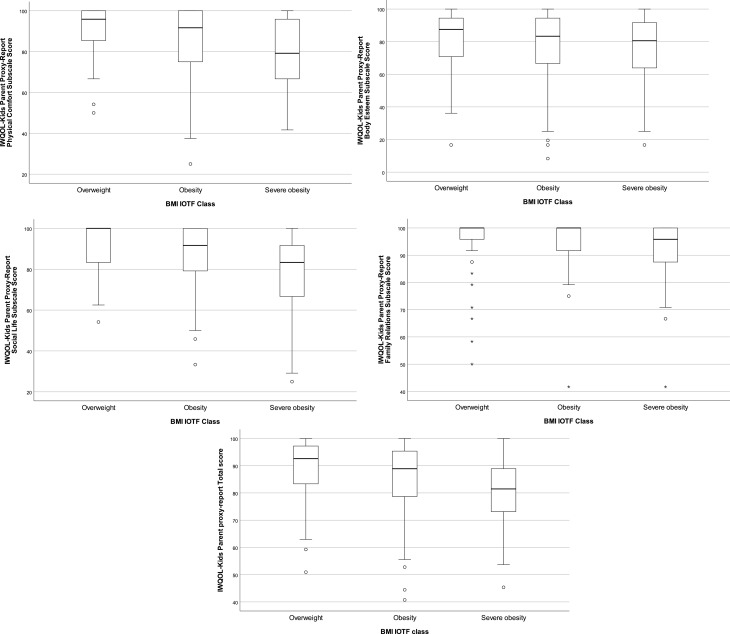
Boxplots of IWQOL-KIDS parent report total and subscale scores per IOTF BMI class

Table 5 shows average total, subscale and item scores on the parent proxy-reported weight-specific HRQOL and whether a significant association with IOTF BMI class was present. Total and subscale scores (p < 0.05), as well as some of the weight-specific HRQOL item scores of the domains physical comfort and social life, were negatively associated with IOTF BMI class (Bonferroni adjusted p < 0.0019). Figure 2 and Supplementary Table 2 show the differences in total and subscale IWQOL-Kids proxy-report scores between IOTF BMI classes. All total and subscale scores differed significantly between the groups with overweight and severe obesity, as well as between groups with obesity and severe obesity, adjusted for age and sex (p < 0.05), except for the body esteem subscale, which did not differ between groups with obesity and with severe obesity. Also, 9% (n = 34) of parents reported that their child did not perceive any impact on weight-specific HRQOL.

### Sensitivity analysis for the IWQOL-Kids

The reliability in the subset of our sample aged ≥ 11 years was similar to the total sample, except for the family relations subscale (parent proxy-report). The age was unknown of 133 children that completed the IWQOL-Kids child-report and 92 children of whom the parents completed the IWQOL-Kids parent proxy-report. These were not included in the age group comparisons. Differences in child-report for age < 11 (n = 31) vs. ≥ 11 (n = 153) are α: total score 0.84 vs. 0.81; physical comfort 0.74 vs. 0.80; body esteem 0.92 vs. 0.92; social life 0.90 vs. 0.87; family relations: 0.84 vs. 0.77. Differences in parent report for age < 11 (n = 188) vs. ≥ 11 (n = 104) are α: total score 0.76 vs. 0.78; physical comfort 0.75 vs. 0.78; body esteem 0.88 vs. 0.91; social life 0.84 vs. 0.87; family relations: 0.57 vs. 0.78.

Supplementary Tables 4 and 5 display the absolute scores of the age group for which the IWQOL-Kids was validated, i.e. ≥11 years. In this smaller sample (child report N = 153, parent report N = 104), scores are slightly lower and only one item score remained significantly associated with the IOTF BMI class on a continuous scale, corrected for age and sex (Bonferroni adjusted p-value ≤ 0.0019). When performing multiple regression analyses with dummy coding to test weight class differences for the IWQOL-Kids parent proxy-report total and subscale scores, most differences remained. Children with severe obesity had significantly lower weight-specific total HRQOL scores than children with obesity or overweight; children with severe obesity had significantly lower scores on physical comfort and social life than children with overweight; and children with severe obesity had significantly lower scores on social life and family relations, as well as a trend toward a significantly lower score (p = 0.07) on physical comfort than children with obesity.

## Discussion

Our results suggest that children (5–19 years) under treatment for overweight, obesity or severe obesity in the Netherlands experience difficulties on all domains of generic and weight-specific HRQOL, such as physical discomfort, social life, feelings, body esteem and school. IOTF BMI class was significantly associated with item scores of the PedsQL subscales physical and social functioning and the IWQOL-Kids subscales physical comfort and social life, reported by their parents. In addition, children with severe obesity have lower generic and weight-specific total and subscale HRQOL scores than children with obesity or overweight, except for scores on emotional functioning and body esteem, which did not differ between children with obesity and those with severe obesity. Interestingly, children with overweight reported similar HRQOL scores as children with obesity. Moreover, PedsQL total and subscale score differences between the IOTF severe obesity class and obesity or overweight class (presented in Supplementary Tables 1 and 2) are larger than minimal clinically important difference scores (MCIDs) [18], except for score differences between severe obesity and obesity classes for emotional and school functioning. Unfortunately, MCIDs are unknown for the IWQOL-KIDS parent proxy-report version. Although only few children (1%) and parents (9%) reported no impact on any of the generic and weight-specific HRQOL items, this percentage was substantially higher for the subscale family relations: 53% of children and 58% of parents reported perceiving no difficulties. Differences remained significant between children with overweight and those with severe obesity when analyzing the subsample aged 11–19, but a linear association with IOTF BMI class as continuous variable disappeared for most IWQOL-Kids parent proxy-report scores.

These results indicate that children with severe obesity (and their parents) report lower generic and weight-specific HRQOL scores than children with overweight or children with obesity. Previous studies report similar findings for generic HRQOL total and subscale scores [2, 18, 19, 21] and for weight-specific HRQOL total and subscale scores [20, 22]. Children with severe obesity are likely to have comorbidities that negatively affect their HRQOL [22, 35], which might explain part of the findings. Although the timing of current data collection preceded the start of a new treatment, past treatment failure could have negatively affected the HRQOL of children with severe obesity more than that of children with overweight or obesity. The lack of a perceived difference in HRQOL between children with overweight and children with obesity (and their parents) found in our study is in line with some studies, but contrasts with most research, which has found differences in HRQOL between children with overweight and children with obesity [2]. This could be explained by the 13% Dutch pediatric prevalence of overweight and obesity, lower than that of other countries (Australia: 25%, Canada: 26%, USA: 33%): the impact of having overweight might thus be greater in the Netherlands, where children with overweight would stand out more. Accordingly, average generic and weight-specific HRQOL scores from our total sample were comparable with scores in clinical samples of youth with obesity/severe obesity from the USA and Australia [18, 36]. Hence not only for children with severe obesity, but also for those with overweight or obesity it is important to pay attention to HRQOL besides weight [8, 9].

Part of the generic HRQOL scores were comparable to the weight-specific HRQOL scores: children and parents reported having difficulties in physical, emotional and social functioning. However, the weight-specific HRQOL scores provided more information on existing obesity-specific problems (such as having difficulty buying clothing, being ashamed of one’s body and having trouble crossing one’s legs) or things that are not impacted by their weight (like family relations). Unfortunately, we only had paired IWQOL-Kids and PedsQL child self-report data of a small highly selected subsample. Accordingly, we were unable to calculate meaningful correlations between generic and weight-specific HRQOL-scores. Previous evidence however suggests that the IWQOL-Kids [8, 9, 14] provides more information about specific difficulties faced by children with overweight, obesity and severe obesity. The IWQOL-Kids is also suggested to be better at detecting differences in HRQOL after a lifestyle intervention [14]. Accordingly, information obtained by this questionnaire can be very useful when setting up, adjusting and evaluating a treatment plan, although the exact timing and choice of measure to assess this PROM needs to be tailored to each patient [37].

### Strengths and limitations

This study had some limitations. First, weight class was unknown for a substantial portion of children, although all children were referred because of their weight, so we can confirm that all included children have a BMI above healthy cut-off levels. The number of missings in our database results from the way participants were recruited. A large part of the data was collected in regular care, where children were not always weighed at the same moment that HRQOL questionnaires were administered. For ethical reasons, children were not weighed solely for purposes of this study. Second, parent proxy- and child-reports, and PedsQL and IWQOL-Kids scores could not be compared due to the small sample size of subgroups resulting from differences in clinical protocols – i.e. the questionnaire used to assess generic and weight-specific HRQOL differed between cohorts. This is unfortunate because studies indicate that parents and children report HRQOL differently [38], and we were unable to take this into account. For pragmatic reasons the IWQOL-Kids was also used for children aged < 11 at some locations. Although validity and reliability are not known for younger children, as far as we know there is no evidence that these scores are invalid, and reliability in our sample was comparable for children under or over age 11. Third, data was collected in 3 different cohorts at 27 locations, sometimes with a very small sample size, hence the effect of location on HRQOL could not be investigated properly. Although associations in observational data might be mediated by factors not included in the analyses, we have minimized these chances by using multiple regression analyses, taking confounders into account, and performing sensitivity analyses.

This study also has strengths. It is the first to investigate both generic and weight-specific HRQOL in Dutch children with overweight, obesity and severe obesity, comparing these groups on total, subscale and item scores. Second, a large group of children with severe obesity was included. Last, by focusing on children that are under care, the results are especially relevant for healthcare settings in which these questionnaires are increasingly used on a regular basis.

### Conclusion and recommendations

In this clinical cohort of Dutch children with overweight, obesity and severe obesity lower scores were found on all generic and weight-specific HRQOL domains, especially physical and social functioning. Children with severe obesity report significantly lower HRQOL scores due to their weight than children with overweight or obesity. Evidence from this study and previous studies suggests that it can be recommended to measure HRQOL, besides weight, in children with overweight, obesity or severe obesity, to tailor the intervention to the child and to evaluate the effects of the intervention. To this end, judging the scores on the questionnaires at the item level provides additional information. Future studies should analyze correlations between paired PedsQL and IWQOL-Kids scores and how these differ between different weight classes.

## Electronic supplementary material

Below is the link to the electronic supplementary material.

Supplementary Material 1

## Data Availability

The datasets generated and analyzed during the current study are not publicly available, but can be obtained from the corresponding author upon reasonable request. Due to the presence of indirect identifiers it is impossible for patients of certain subgroups to stay completely anonymous.

## Abbreviations

BMI: body mass index
HRQOL: health-related quality of life
IOTF: International Obesity Task Force
IWQOL-Kids: Impact of Weight on Quality Of Life for Kids
MCIDs: minimal clinically important difference scores
PedsQL: Pediatric Quality of Life Inventory
PROM: patient-reported outcome measure
SDS: standard deviation scores
