# Supplementary material for: Health-related quality of life in children under treatment for overweight, obesity or severe obesity: a cross-sectional study in the Netherlands
Source: BMC Pediatr. 2023 Apr 11;23:167. doi: 10.1186/s12887-023-03973-8 (PMC10088296; doi:10.1186/s12887-023-03973-8)
Supplement: Supplementary file 1 — Supplementary Material 1 [file 12887_2023_3973_MOESM1_ESM.docx]

**Supplementary Table 1. Average total and subscale scores for the PedsQL child report per IOTF BMI class**

|  | **Overweight** N=97 | | **Obesity** N=120 | | **Severe obesity** N=153 | |
| --- | --- | --- | --- | --- | --- | --- |
|  | **Mean** | **SD** | **Mean** | **SD** | **Mean** | **SD** |
| **Physical functioning** | 81.2 | 13.7 | 80.6 | 15.7 | 70.8 ^1,2^ | 17.4 |
| **Emotional functioning** | 76.0 | 18.0 | 72.2 | 23.6 | 67.6 ^1^ | 22.1 |
| **Social functioning** | 83.3 | 17.0 | 81.5 | 18.9 | 71.6 ^1,2^ | 21.9 |
| **School functioning** | 78.7 | 17.3 | 75.4 | 16.6 | 68.7 ^1,3^ | 20.3 |
| **Total score** | 80.0 | 12.6 | 77.8 | 15.3 | 69.8 ^1,2^ | 16.6 |

^1^Significant difference between groups with overweight and severe obesity, adjusted for age and sex (p<0.05)
^2^Significant difference between groups with obesity and severe obesity, adjusted for age and sex (p<0.05)
^3^Trend toward a significant difference between groups with obesity and severe obesity, adjusted for age and sex (p<0.10)

**Supplementary Table 2. Average total and subscale scores for the IWQOL-Kids parent report per IOTF BMI class**

|  | **Overweight** N=97 | | **Obesity** N=116 | | **Severe Obesity** N=68 | |
| --- | --- | --- | --- | --- | --- | --- |
|  | **Mean** | **SD** | **Mean** | **SD** | **Mean** | **SD** |
| **Physical comfort** | 91.0 | 12.2 | 86.0 | 15.4 | 80.3^1,2^ | 16.3 |
| **Body esteem** | 81.7 | 18.1 | 77.8 | 21.2 | 74.6^1^ | 21.2 |
| **Social life** | 90.5 | 13.2 | 87.1 | 15.1 | 79.8^1,2^ | 18.0 |
| **Family relations** | 95.0 | 10.1 | 95.4 | 8.2 | 91.0^1,2^ | 11.4 |
| **Total score** | 88.9 | 10.8 | 85.6 | 13.1 | 80.6^1,2^ | 13.2 |

### ^1^Significant difference between groups with overweight and severe obesity, adjusted for age and sex (p<0.05) ^2^Significant difference between groups with obesity and severe obesity, adjusted for age and sex (p<0.05)

**Supplementary Table 3 Average total, subscale and item scores for the PedsQL child report for the subgroup ≥8 years (n=363)**

|  | **Mean** | **SD** | **P-Value** |
| --- | --- | --- | --- |
| **Physical functioning** | **78.5*** | **16.0** | **<0.001** |
| 1. Hard to walk more than one block | 76.7* | 29.8 | <0.001 |
| 1. Hard to run | 68.5* | 31.6 | <0.001 |
| 1. Hard to do sports or exercises | 73.5* | 27.5 | <0.001 |
| 1. Hard to lift something heavy | 76.7 | 26.3 | 0.377 |
| 1. Hard to take bath or shower | 92.9 | 18.8 | 0.997 |
| 1. Hard to do chores around the house | 82.0 | 24.9 | 0.062 |
| 1. Hurth or aches | 70.4 | 27.3 | 0.097 |
| 1. Low energy | 72.0* | 26.6 | <0.001 |
| **Emotional functioning** | **69.3*** | **21.4** | **0.012** |
| 1. Feel afraid or scared | 66.3 | 27.8 | 0.323 |
| 1. Feel sad or blue | 65.2 | 29.9 | 0.049 |
| 1. Feel angry | 66.3 | 28.8 | 0.029 |
| 1. Trouble sleeping | 76.1 | 34.9 | 0.039 |
| 1. Worry about what will happen | 72.8 | 31.9 | 0.030 |
| **Social functioning** | **75.3*** | **25.4** | **<0.001** |
| 1. Trouble getting along with peers | 80.4 | 30.1 | 0.576 |
| 1. Other kids not wanting to be friend | 73.9 | 34.9 | 0.108 |
| 1. Teased | 61.4* | 35.1 | <0.001 |
| 1. Doing things other peers do | 78.3 | 29.5 | 0.006 |
| 1. Hard to keep up when play with others | 81.5***** | 29.5 | 0.002 |
| **School functioning** | **73.8*** | **18.9** | **0.005** |
| 1. Hard to concentrate | 83.7 | 28.8 | 0.204 |
| 1. Forget things | 62.0 | 34.4 | 0.325 |
| 1. Trouble keeping up with schoolwork | 81.5 | 27.5 | 0.020 |
| 1. Miss school – not well | 78.3 | 27.5 | 0.040 |
| 1. Miss school – doctor appointment | 69.3 | 24.3 | 0.007 |
| **Total score** | **75.2*** | **15.9** | **<0.001** |
| * Significant association with IOTF BMI class, adjusted for age and sex (<0.05). For item scores a Bonferroni adjusted p-value 0.05/23=0.0022 was considered statistically significant.  † For item scores a trend toward a significant association with IOTF BMI class, adjusted for age and sex (Bonferroni adjusted p-value 0.10/23=0.0043) | | |  |

**Supplementary Table 4 IWQOL-Kids child report scores for the subgroup ≥11 years**

| **IWQOL-Kids** | **Child report** N=153 | |
| --- | --- | --- |
|  | **Mean** | **SD** |
| **Physical comfort** | **71.8** | **20.4** |
| 1. Avoid stairs | 73.9 | 28.1 |
| 1. Hard to bend over | 73.2 | 29.0 |
| 1. Hard to move around | 73.8 | 26.6 |
| 1. Hard to fit into seats in public places | 86.0 | 23.5 |
| 1. Knees or ankles hurt | 69.3 | 29.6 |
| 1. Hard to cross legs | 52.8 | 36.5 |
| **Body esteem** | **57.2** | **27.3** |
| 1. Ashamed of body | 42.0 | 31.2 |
| 1. Don’t like myself | 68.8 | 33.8 |
| 1. Try not to look at myself in mirrors or photographs | 73.4 | 32.5 |
| 1. Hard time believing compliments | 60.9 | 35.4 |
| 1. Lack in self-confidence | 58.3 | 35.8 |
| 1. Avoid activities that involve wearing shorts/bathing suits | 57.0 | 36.6 |
| 1. Difficult to buy clothing | 41.2 | 34.3 |
| 1. Don’t like to undress in front of others | 48.5 | 36.5 |
| 1. Embarrassed to try out for activities at school | 63.0 | 35.5 |
| **Social life** | **77.6** | **20.6** |
| 1. Teased of made fun of | 68.8 | 28.9 |
| 1. People talk about me behind my back | 67.7 | 28.3 |
| 1. People avoid spending time with me | 86.1 | 23.1 |
| 1. People stare at me | 68.8 | 31.0 |
| 1. Trouble making/keeping friends | 84.6 | 25.6 |
| 1. People think that I am not smart | 87.8 | 22.3 |
| **Family relations** | **91.8** | **13.2** |
| 1. Family members treat me differently | 87.2 | 23.7 |
| 1. Family member talk behind my back | 91.6 | 20.1 |
| 1. Family members reject me | 95.6 | 14.5 |
| 1. My parents are not proud of me | 93.3 | 17.3 |
| 1. Family members make fun of me | 87.8 | 21.3 |
| 1. Family members don’t want to be seen with me | 94.6 | 17.8 |
| **Total score** | **72.8** | **17.4** |

**Supplementary Table 5 IWQOL-Kids parent report scores for the subgroup ≥11 years**

| **IWQOL-Kids** | **Parent-report** N=104 | | |
| --- | --- | --- | --- |
|  | **Mean** | **SD** | **P-Value** |
| **Physical comfort** | **83.9*** | **16.1** | **0.046** |
| 1. Avoid stairs | 82.8 | 25.0 | 0.796 |
| 1. Hard to bend over | 79.4 | 26.6 | 0.082 |
| 1. Hard to move around | 76.4 | 25.8 | 0.274 |
| 1. Hard to fit into seats in public places | 93.0 | 16.4 | 0.029 |
| 1. Knees or ankles hurt | 86.7 | 21.5 | 0.100 |
| 1. Hard to cross legs | 84.2 | 24.0 | 0.137 |
| **Body esteem** | **75.7** | **21.1** | **0.657** |
| 1. Ashamed of body | 64.5 | 30.9 | 0.564 |
| 1. Don’t like myself | 81.4 | 25.1 | 0.623 |
| 1. Try not to look at myself in mirrors or photographs | 87.0 | 21.6 | 0.188 |
| 1. Hard time believing compliments | 79.0 | 25.7 | 0.972 |
| 1. Lack in self-confidence | 74.5 | 31.4 | 0.602 |
| 1. Avoid activities that involve wearing shorts/bathing suits | 78.6 | 25.9 | 0.065 |
| 1. Difficult to buy clothing | 63.0 | 31.7 | 0.673 |
| 1. Don’t like to undress in front of others | 68.2 | 33.4 | 0.631 |
| 1. Embarrassed to try out for activities at school | 79.4 | 25.2 | 0.341 |
| **Social life** | **83.1†** | **16.9** | **0.094** |
| 1. Teased of made fun of | 75.0 | 25.7 | 0.864 |
| 1. People talk about me behind my back | 75.9 | 25.1 | 0.615 |
| 1. People avoid spending time with me | 88.0 | 19.5 | 0.319 |
| 1. People stare at me | 83.3* | 21.1 | <0.001 |
| 1. Trouble making/keeping friends | 85.8 | 21.1 | 0.155 |
| 1. People think that I am not smart | 89.7 | 17.7 | 0.155 |
| **Family relations** | **93.0** | **11.7** | **0.707** |
| 1. Family members treat me differently | 92.0 | 18.3 | 0.334 |
| 1. Family member talk behind my back | 90.8 | 18.8 | 0.741 |
| 1. Family members reject me | 95.2 | 15.0 | 0.600 |
| 1. My parents are not proud of me | 94.7 | 14.9 | 0.763 |
| 1. Family members make fun of me | 86.3 | 22.5 | 0.537 |
| 1. Family members don’t want to be seen with me | 98.1 | 8.8 | 0.012 |
| **Total score** | **83.1** | **13.5** | **0.157** |

* Significant association with IOTF BMI class, adjusted for age and sex (p≤0.05). For item scores a Bonferroni adjusted p- value 0.05/27=0.0019 was considered statistically significant.

**†** Trend toward a significant association with IOTF BMI class, adjusted for age and sex (p≤0.10).
